# Shared Decision-Making With a Virtual Patient in Medical Education: Mixed Methods Evaluation Study

**DOI:** 10.2196/22745

**Published:** 2021-06-10

**Authors:** Simon Jacklin, Neal Maskrey, Stephen Chapman

**Affiliations:** 1 School of Pharmacy and Bioengineering Keele University Keele United Kingdom

**Keywords:** shared decision making, virtual patient, communication, medical education

## Abstract

**Background:**

Shared decision-making (SDM) is a process in which clinicians and patients work together to select tests, treatments, management, or support packages based on clinical evidence and the patient’s informed preferences. Similar to any skill, SDM requires practice to improve. Virtual patients (VPs) are simulations that allow one to practice a variety of clinical skills, including communication. VPs can be used to help professionals and students practice communication skills required to engage in SDM; however, this specific focus has not received much attention within the literature. A multiple-choice VP was developed to allow students the opportunity to practice SDM. To interact with the VP, users chose what they wanted to say to the VP by choosing from multiple predefined options, rather than typing in what they wanted to say.

**Objective:**

This study aims to evaluate a VP workshop for medical students aimed at developing the communication skills required for SDM.

**Methods:**

Preintervention and postintervention questionnaires were administered, followed by semistructured interviews. The questionnaires provided cohort-level data on the participants’ views of the VP and helped to inform the interview guide; the interviews were used to explore some of the data from the questionnaire in more depth, including the participants’ experience of using the VP.

**Results:**

The interviews and questionnaires suggested that the VP was enjoyable and easy to use. When the participants were asked to rank their priorities in both pre- and post-VP consultations, there was a change in the rank position of *respecting patient choices*, with the median rank changing from second to first. Owing to the small sample size, this was not analyzed for statistical significance. The VP allowed the participants to explore a consultation in a way that they could not with simulated or real patients, which may be part of the reason that the VP was suggested as a useful intervention for bridging from the early, theory-focused years of the curriculum to the more patient-focused ones later.

**Conclusions:**

The VP was well accepted by the participants. The multiple-choice system of interaction was reported to be both useful and restrictive. Future work should look at further developing the mode of interaction and explore whether the VP results in any changes in observed behavior or practice.

## Introduction

### Background

Shared decision-making (SDM) is a process in which clinicians and patients work together to select tests, treatments, management, or support packages based on clinical evidence and the patient’s informed preferences [1]. The General Medical Council, National Institute for Health and Care Excellence, and National Health Service England all recognize that SDM should become the norm for clinical practice. This is supported by the Montgomery ruling, which provides a legal basis for SDM [2] and established that rather than a clinician deciding what they think a patient should be told, patients should be told whatever they would like to know [3]. In addition to ethical and legal arguments, SDM has been shown to improve patient satisfaction [4], decrease decisional conflict [5], and reduce antibiotic prescription [6].

The Care Quality Commission 2018 annual survey of National Health Service hospital inpatients [7] included a question that asked, “Were you involved in decisions about your care as much as you wanted to be?” Of the patients who responded, 11% answered “No” and 35% answered “Yes, to some extent,” suggesting that SDM did not occur to the optimal extent.

Professionals have been found to consciously adopt a paternalistic decision-making style to care for their patients, as they feel that their knowledge and experience enables them to make decisions in the patients’ best interests [8,9]. Mulley et al [10] refer to this as the *silent misdiagnosis*, because if patients are not involved in decisions about their care, they cannot communicate what outcomes matter to them as individuals and thus which course of action may be the most appropriate.

There are many barriers to the wider adoption of SDM [11]. Some of these, such as longer appointment times, require system-level interventions to resolve, but others are concerned with individual practitioners. One such barrier is professionals having the skills embedded so that SDM becomes routine within their practice. SDM represents a new approach to patient care, which requires a set of consultation skills that may differ from those currently used by professionals [12].

The amount of time dedicated to consultation skills in undergraduate medical education varies and has been found to be as low as 0.15% of the curriculum time [13]. The level of SDM within undergraduate medical education is unclear but a review of the literature suggests that it is low [14]. The focus in postgraduate medical education varies based on specialty but some feature very little variation that is unwarranted [15,16]. The teaching of consultation skills is often confined to the first few years of medical undergraduate courses, and the subject may be taught separately rather than fully integrated with other clinical content. This does not reflect optimum clinical practice as described by the General Medical Council and could frame consultation skills as something less important than other more knowledge-based areas of the curriculum.

SDM is a skill [17], and all skills require deliberate practice and feedback to be acquired and improved [18]. In the context of SDM, any practice often uses simulated patients (SPs), role-plays with peers or with actors, or real patients, all of which have issues associated with their use. These include poor-quality acting, lack of standardization, and resource intensity [19,20]. In such environments, opportunities for learners to repeat their consultation skills or test different approaches to a consultation for themselves are limited and usually not possible.

Virtual patients (VPs) are a “specific type of computer program that simulates real-life clinical scenarios; learners emulate the roles of health care providers to obtain a history, conduct a physical exam, and make diagnostic and therapeutic decisions” [21]. In contrast to other traditional and widely used approaches to practicing consultation skills, VPs may offer a method that is standardized, customizable, repeatable, flexible, low risk, and accessible at any time to a large number of learners.

### Objective

This study aims to evaluate the views of undergraduate medical students toward a VP workshop aimed at developing the skills required for SDM.

## Methods

### Population

The Manchester Medical Research Student Society holds an annual student conference. SC was invited to run an educational session using a VP. Medical student delegates attended the session voluntarily, and it was from this session that the participants were recruited. Participation in the study was voluntary.

### Intervention

**Figure 1:**
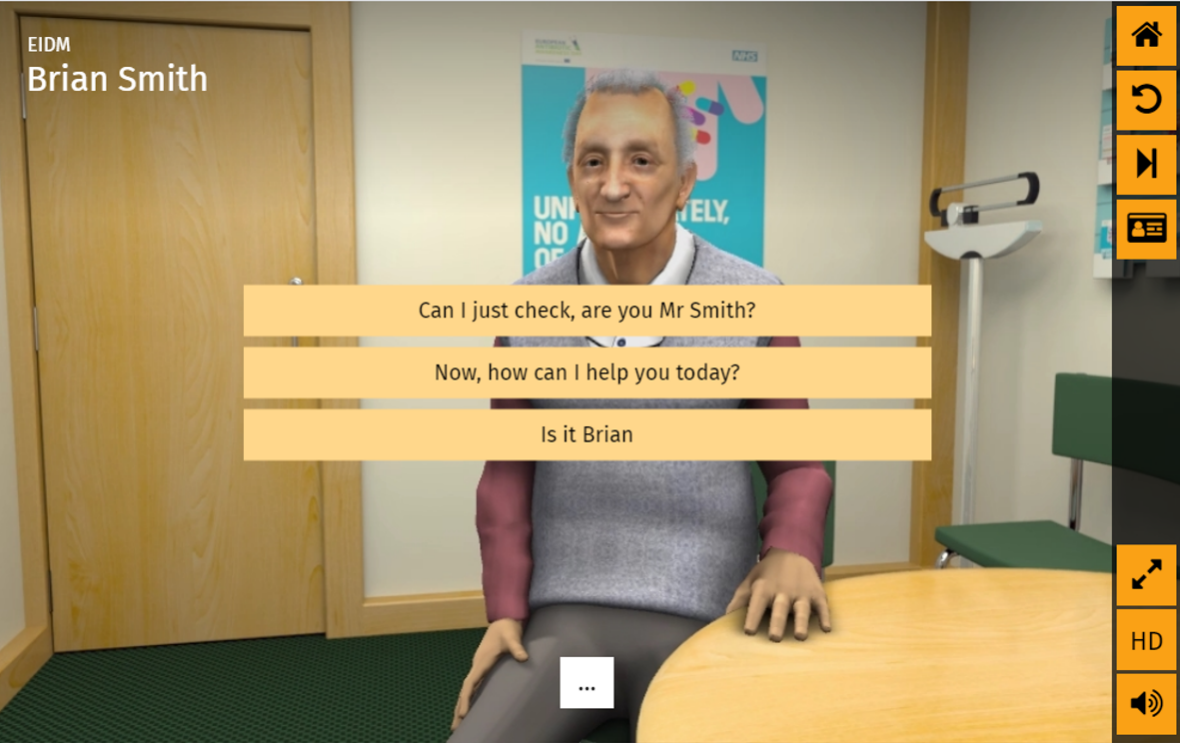
Screenshot of the virtual patient.

The intervention was a VP that simulated a single primary care consultation. The VP, Brian Smith, comes to discuss whether to initiate a statin after referral from the practice nurse. The VP was accessible from a website and usable on multimedia devices. Interaction with the VP was achieved via multiple-choice selection, and personalized feedback was delivered at the end of the simulation. The design process for the VP was previously published [22] and a screenshot is provided in Figure 1.

### Evaluation Process

The setting for the evaluation was a 1-hour clinical decision-making workshop at a medical student conference. A mixed methods evaluation focusing on the VP component of the workshop was conducted.

All students were provided with information sheets and consent forms and could decide whether they wished to take part. A total of 22 participants completed a consent form and prequestionnaire immediately before using the VP and then completed a postquestionnaire immediately afterward; the students were given 30 minutes to use the VP independently on their own or a borrowed device. This gave the students the opportunity to run through the consultation multiple times. Shortly after the workshop, participants who completed the questionnaire evaluation were emailed to invite them to participate in a semistructured interview; a £10 (US $14) Amazon voucher was offered to participants who consented to an interview to compensate them for their time. The interviews were planned to use purposive sampling, but ultimately, a convenience sample was used because of low recruitment. The questionnaire provided cohort-level data and helped to inform the interview guide; the interviews were used to explore some of the data from the questionnaire in more depth. The interview asked the participants about their experience of the VP, how useful it was for developing communication skills required for SDM, and at which point in the medical curriculum the VP might be best placed.

Ethical approval was obtained from the Keele University Faculty of Health Ethical Review Panel.

### Data Analysis

#### Overview

Quantitative data were analyzed using descriptive statistics. A single question asked the participants to rank their treatment priorities during a consultation both before and after using the VP; this was based on the prescribing principles proposed by Barber [23].

The qualitative data were obtained from semistructured interviews conducted by SJ over the telephone. None of the participants knew SJ before the study. The data were analyzed using semantic thematic analysis, with codes derived from raw data, not from preexisting theory. The process described by Braun and Clarke [24] was used and is outlined below.

#### Step 1: Data Familiarization

The transcript was read over while listening to the audio recording. This had the dual function of checking the transcript for accuracy and familiarizing the coder with the data.

#### Step 2: Generation of Initial Codes

The transcripts were coded using NVivo 11 (QSR International) using what Braun and Clarke [24] called semantic coding; the surface meaning of the words used by the participants was of interest, rather than trying to identify the features that resulted in the form and meaning of the words as in latent coding.

#### Step 3: Searching for Themes

After the transcripts had been annotated with codes, the codes were grouped together into overarching themes.

#### Step 4: Reviewing Themes

The initial themes were refined by combing themes or leaving certain themes that lacked support from the data.

#### Step 5: Defining Themes

This step involved clarifying what each theme captured and why it was important to go beyond just paraphrasing the data.

#### Step 6: Write Up

Once the final themes had been established, the report was written.

NVivo 11 (QSR International) was used to organize the coding. SJ was the only coder; however, the codes and themes were discussed with SC and NM to encourage reflexivity. Member checking was not undertaken because of the power imbalance present between a participant and the researcher; a participant may well acquiesce to the researchers’ suggestions, thus giving a false impression of validity [25].

## Results

### Questionnaire Data

A total of 24 students participated in the workshop, and 22 participated in the study by completing both the prequestionnaires and postquestionnaires; 2 students declined to participate in the study. Table 1 presents the demographic data of the participants in the questionnaire phase of the study.

**Table 1:** Demographic data of participants in the questionnaire phase.

Characteristics			Participants, n (%)
**Gender**			
	Male	6 (27)	
	Female	16 (73)	
**Year of study**			
	First	1 (5)	
	Second	5 (23)	
	Third	0 (0)	
	Fourth	8 (36)	
	Fifth	7 (32)	
	Sixth	1 (5)	

Most participants found the VP enjoyable to use, with 19 of them suggesting that it was either “enjoyable” or “very enjoyable” to use. They also found it accessible, with 100% (22/22) of the study participants rating it as either “very accessible” or “accessible.”

Table 2 shows a distribution of views on the format of the reply, the multiple-choice interaction system.

**Table 2:** Participants’ views on the format of reply to the virtual patient.

Evaluation of reply format^a^	Respondents, n (%)
1	0 (0)
2	4 (18)
3	5 (23)
4	10 (45)
5	3 (14)

^a^Scores range from 1, very poor, to 5, very good.

As Table 3 shows, just over half (13/22, 59%) of the participants suggested that it was “likely” or “highly likely” that there would be a change in their practice as a result of using the VP. Most of the changes suggested were related to either being more patient centered or trying to engage in SDM.

**Table 3:** Participants’ self-reported likelihood of change in their clinical practice.

Likelihood of change	Respondents, n (%)
Highly unlikely	2 (9)
Unlikely	7 (32)
Likely	11 (50)
Highly likely	2 (9)

The participants were asked to rank four priorities, without the possibility of equal rankings. When comparing the preintervention responses with the postinterview responses, there was a change in the rank position of “respecting patient choices,” shifting from a median position of second to first (Table 4).

**Table 4:** Participants’ priorities during a consultation, preintervention and postintervention.

Priorities within a consultation		Respondents, n (%)			
		Fourth	Third	Second	First
**Preintervention**					
	Maximizing effectiveness	0 (0)	8 (36)	8 (36)	6 (27)
	Minimizing risks	0 (0)	7 (32)	9 (41)	6 (27)
	Minimizing costs	22 (100)	0 (0)	0 (0)	0 (0)
	Respecting patient choices	0 (0)	7 (32)	5 (23)	10 (45)
**Postintervention**					
	Maximizing effectiveness	0 (0)	12 (55)	6 (27)	4 (18)
	Minimizing risks	0 (0)	7 (32)	10 (45)	5 (23)
	Minimizing costs	22 (100)	0 (0)	0 (0)	0 (0)
	Respecting patient choices	0 (0)	3 (14)	6 (27)	13 (59)

### Interview Data

A total of 7 participants consented to an interview; all of them were interviewed. A total of 3 main themes were constructed from the interview transcript data. These themes are elaborated here using verbatim quotations. Data saturation was not reached as the last interview resulted in subtle restructuring of the themes; the major themes were established after the sixth interview. The major themes were as follows:

- Bridging: the VP was suggested to be useful in helping medical students transition from preclinical to clinical teaching in undergraduate studies.
- Exploring the consultation: the VP permitted the user to explore different approaches during a consultation, something that is difficult to do in conversation with simulated or real patients.
- Personal style and subjectivity: every doctor has their own style for consulting with patients, and it was suggested that the VP did not reflect this.

### Bridging

The theme bridging describes the VP helping learners transition from one part of the undergraduate course to another. Specifically, some participants suggested that when one first encounters patients or actors in an undergraduate course, the experience is intimidating and overwhelming. Some of the participants suggested that the VP could be usefully deployed between the early, theory-based years of the course and the later, more patient-oriented ones to act as a stepping stone:

I think that’s where it has a lot of value because I know that there are quite a lot of people in Medical School who start off Medical Schoolby doing quite scientifical [sic] things and then when it gets to their first patient contact, it can be very daunting and it can be quite frightening because you don’t really know what to say. People can teach you how to take a history but if you’re sat in front of someone and you have to chat to them for bit, then it can feel quite awkward to start off with. I think if you had some kind of virtual introduction to all of this, it can make things a bit easier when you actually get into it.

P3

A key part of the reason why the VP was useful as a bridge related to the multiple-choice response system. As there were multiple options presented each time, the participants could read them and receive a prompt, a suggestion of how to phrase something. This was posited as useful for learning, particularly in the earlier years of undergraduate study:

I definitely think that would be useful, like getting those prompts of what’s good to say [yeah] when you’re starting clinical years, I think would be, personally I would’ve found that really helpful because that’s something that takes a while to pick up and you sort of learn, I think you learn more from seeing other people do it and hearing other people do it. So, if virtual patient can be those prompts for you and you can learn from that, then I think that would be really helpful.

P2

### Exploring the Consultation

The participants suggested that the VP allowed them to explore the consultation. By using the VP multiple times, the participants reported that they were able to try different routes, phrases, or approaches to the consultation. The demonstration of the consequences of one’s actions seemed to be beneficial to the exploration. This may form a part of *bridging* but is perhaps also a separate theme:

It’s probably good in terms of it makes you more likely to explore different ways of managing a situation. Some might be wrong; some might be right but even if you take the wrong route, nothing serious is going to happen at the end of the day. It’s not like you’ve committed an offence or anything like that. I think it’s quite good in terms of that.

P3

I think what I quite like about the virtual one is that you can take it in different directions and almost test it out. Sometimes it’s harder to do that with a simulated patient just because you don’t get the opportunity to do it again.

P7

The second of these responses (P7) contrasts the VP with SPs, suggesting that the latter do not permit one to explore a consultation to the same degree as a VP. The idea that the VP allowed participants to repeatedly explore a consultation in a manner that an SP does not was further expanded

I think if you learn from your mistakes and do it again and that’s what it’s good for as well because with a simulated patient, you can stop and start. When we have the Simulated Patient Workshops, if you’re stuck or if you don’t know where to go next, you can always stop and it’s quite a safe space but you can never really just take the whole thing and start all over again because there’s a schedule that you have to go with. You only have a certain amount of time. You can only do one scenario because there are loads of people that need to go through. At least with something like that, it kind of releases the tension because you can just do it over and over again. You don’t have that extra time management problem.

P3

This response suggests that one benefit that the VP had over an SP was that time was not an issue. The VP gave the students a greater amount of time to practice, and they could explore the consultation multiple times.

### Personal Style and Subjectivity

The multiple-choice system of interaction was a feature of the VP that divided participants’ opinions. For some participants, it provided a useful prompt of phrases they could use (*Bridging* section), whereas for others, it was not flexible enough to encompass their own personal consultation style:

I guess the obvious thing was that you are limited by what you can say. You have to choose [from] the answers which the computer gives.

P4

I think I thought some of the stuff was a good prompt as to like, you know, I should be saying this or I should have spoken about this.

P5

Erm, obviously everyone has their own flow and way of doing consultations [mmm] erm and the algorithm just gave three options, it was really difficult to choose basically, it could be more flexible.

P6

It seems that the restriction experienced by some participants was not just concerned with the three choices presented at each point but the order in which the consultation could be navigated:

*I think I didn’t find them restrictive in the sense that I would chose something else apart from the three options, it’s just that the order of, you know, the consultation, like the order in which the consultation was done, there was no flexibility to it [OK yeah]. So, you’d go from a certain topic first and then you had to move onto another topic and then you’d get the final topic. But their own style might be different,they might have the consultation in a different order [yeah, yes] than you could.* [P5]

## Discussion

### Principal Findings

This study positively evaluated a VP workshop for developing the consultation skills required to engage in SDM with patients. The medical student participants suggested that the simulation was enjoyable to use, easy to access, and there was a change in the participants’ prescribing priorities when comparing pre- and post-VP consultation. The interviews suggested that the VP was useful in allowing students to explore a consultation and trying different phrases and approaches in a consultation to see what effect they had. The results suggested that the VP could be a useful tool to help students progress from the early, theory-focused years of medical school to the more patient-oriented ones later.

When comparing pre- and post-VP responses, there appeared to be a change toward a more patient-centered priority. As Table 4 shows, there was a change in the rank position of “respecting patient choices,” with the median rank changing from second to first. This would seem to be a favorable change, as it reflects the current opinion about the promotion of SDM [26]. There are a few caveats to this measurement. First, there was no analysis of student interactions with the VP; therefore, whether this would translate into a change in practice in clinical situations is unknown. It is also unknown whether any change would endure over time. These points are particularly germane because SDM is suggested to be philosophically valued by professionals but not necessarily practiced [11,27,28].

The theme of *exploring* from the interviews described the important opportunity VPs provide for safe and repetitive exploration of a consultation. As it was not a real person, the participants suggested that they felt at ease trying out different techniques and phrases, exploring the consultation with a variety of approaches. Unlike an SP interaction, the VP was not subject to the same time constraints; therefore, it could be reset and used multiple times.

The interview data suggested that the exploratory nature of the VP meant that it could serve as a useful intervention for bridging from the early, theory-focused years of medical school to the more patient-oriented ones later. Medical students have been reported to find interactions with SPs stressful [29], and VPs have been found to improve learner confidence before interacting with SPs [30] or real patients [31]. This seems to be because the VP is not a real person; therefore, failure did not incur the same consequences as with another human, even when simulating. The VP allowed repetitive, safe practice, which is essential for skill acquisition and improvement [18].

The VP featured a multiple-choice system of interaction where the learner could select one of the responses displayed on the screen; this system was reported to have both positive and negative elements. The potential benefit for the learner, as the interview data suggested, is that the limited but multiple options act as a kind of prompt, suggesting alternate phrases or routes through the consultation. Students have to read the options available to respond; therefore, they are forced, however briefly, to consider a range of responses and potentially explore them. These *prompts* could help teach or remind learners of more or less helpful approaches they could use or not use in real-world consultations.

The main limitation of the multiple choice was its restrictive nature, which meant that it could not incorporate the learners’ individual consultation styles. This sense of restriction was reported as a negative experience by some of the participants, and for them, it also altered the learning experience. This is a recognized issue in the design of multiple-choice VPs [32]. With a free-text VP, where one can type in any preferred response, the learner must recall the phrase they want to use by thinking independently, similar to a real conversation. With a multiple-choice VP, a learner is only required to recognize the *correct* response from the three options presented. However, the VP was designed according to the evidence and principles of a good consultation [22]; therefore, if the learner wanted to take a different action, it could be that they wanted to consult in a way that was not optimal. McCartney et al [12] suggest that SDM requires professionals to consult in new and different ways, and surveys suggest that the teaching of consultation skills and SDM is relatively low in some undergraduate courses [13,14]. It could indeed be the case that the VP was too restrictive to adequately reflect the flexible nature of a consultation; however, there could also be an issue of overconfidence bias leading to learners wanting to consult in their own individual but suboptimal way. The latter point is particularly germane when one considers some of the participants’ responses, which demonstrated that they had not yet fully understood what SDM entails.

The literature suggests that simulation learning mirrors the theory of reflective practice by Kolb [33], where all learning occurs after the simulation through reflection on a concrete experience [34]. This evaluation suggests that for this VP, some learning occurred during the experience, not solely afterward. Thus, the Kolb theory may not apply that well to this VP; instead, the theory by Schon may be a more relevant theoretical approach, as it differentiates between reflection-in-action and reflection-on-action [35].

Educational feedback was an element that was not touched on in this evaluation. Feedback is often overlooked in the VP literature, although some studies have explored this issue [36,37]. Future work could explore how feedback can be delivered and facilitated most effectively with this VP.

A limitation of this evaluation is that the authors of this paper were also the designers of the VP [22]. This introduces a potential bias. Second, there was only a single coder (SJ) for the interview data, introducing another potential source of bias. To reduce the effect of this potential bias, all transcripts and quotes were discussed among the 3 authors to encourage reflexivity. Another source of bias results from the participants attending the workshop voluntarily; the potential participants were self-selecting, as delegates to the conference could choose whether to attend the workshop. Finally, the sample size was limited because of the small number of delegates to the conference and subsequent attendance at the VP workshop. This resulted in data saturation not being reached during the interviews; there is potential for further interviews to change the conclusions of this paper.

The purpose of this study is to report early work on a VP to develop SDM skills. Both the interviews and questionnaires indicate that there is sufficient perceived value in VPs as a training tool to make it worthwhile to develop further. Future work should build on this to form a more complete picture of the application of VP to SDM. This work should include exploring the VP with larger groups of students, focusing on how the VP could be integrated into an undergraduate curriculum and the effect the VP has on students’ subsequent consultations with patients. Further work could also explore the role of these simulations in developing the SDM skills of postgraduate professionals; for example, continuing professional development.

### Conclusions

The VP was found to be accessible and enjoyable; in addition, it made some participants suggest that they would make changes in their practice. The VP also induced a change in participants’ self-reported priorities during a consultation.

The multiple-choice system was suggested to be key to the way the VP worked, prompting the users with ideas of what to say. The participants were all undergraduates; therefore, it is unknown whether postgraduates would require prompting in a simulated conclusion. It is therefore a direction for future research to see whether postgraduate health professionals would find the multiple-choice prompts useful. The multiple-choice system was not universally popular, as some participants felt it restricted them from consulting in their natural way; it is unknown whether their preferred consultation style is in line with best practice and evidence around consultation skills and SDM. Consideration will also be given to using the VP with earlier-year students so that they can experience it before interactions with SPs. This too will require evaluation to observe the effect that these changes may have.

## Abbreviations

SDM: shared decision-making
SP: simulated patient
VP: virtual patient
